# A daily fever curve for the Swiss economy

**DOI:** 10.1186/s41937-020-00051-z

**Published:** 2020-07-09

**Authors:** Marc Burri, Daniel Kaufmann

**Affiliations:** 1 Institute of Economic Research, University of Neuchâtel, Rue A.-L. Breguet 2, Neuchâtel, 2000 Switzerland; 2 KOF Swiss Economic Institute, ETH Zurich, Zurich, Switzerland

**Keywords:** COVID-19, Composite leading indicator, Financial market data, News sentiment, Switzerland

## Abstract

Because macroeconomic data is published with a substantial delay, assessing the health of the economy during the rapidly evolving COVID-19 crisis is challenging. We develop a fever curve for the Swiss economy using publicly available daily financial market and news data. The indicator can be computed with a delay of 1 day. Moreover, it is highly correlated with macroeconomic data and survey indicators of Swiss economic activity. Therefore, it provides timely and reliable warning signals if the health of the economy takes a turn for the worse.

## Introduction

Because macroeconomic data is published with a substantial delay, assessing the health of the economy during the rapidly evolving coronavirus disease of 2019 (COVID-19) crisis is challenging. Usually, policy makers and researchers rely on early information from surveys and financial markets to construct leading indicators and estimate forecasting models (see, e.g., Abberger et al., 2014; Galli, 2018; Kaufmann and Scheufele, 2017; OECD, 2010; Stuart, 2020; Wegmüller and Glocker, 2019, for Swiss applications). These indicators and forecasts are published with a delay of 1 to 2 months.^1^ During the COVID-19 crisis, however, we need high-frequency information to assess how stricter or looser health restrictions and economic stimulus programs affect the economy.

We propose a novel daily fever curve (*f*-curve) for the health of the Swiss economy based on publicly available financial market and news data. We construct risk premia on corporate bonds, term spreads, and stock market volatility indices starting in 2000. In addition, we collect short economic news from online newspaper archives. We then estimate a composite indicator which has the interpretation of a fever curve: As for monitoring the condition of a patient, an increase of the fever curve provides a reliable and timely warning signal if health takes a turn for the worse.

**Fig. 1:**
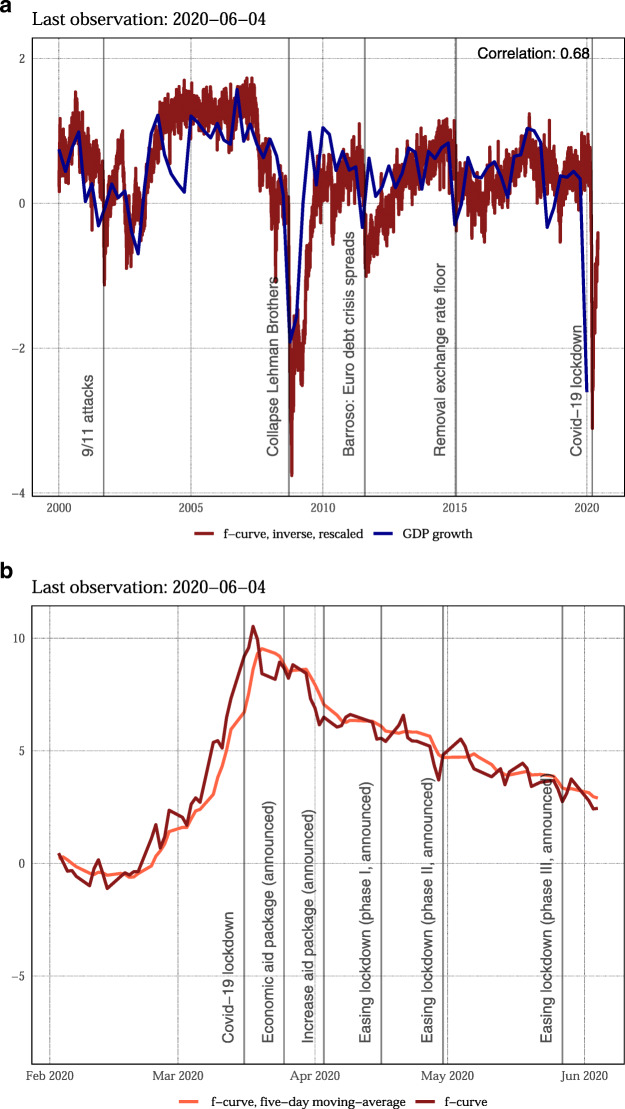
A fever curve for the Swiss economy. Panel **a** compares the fever curve (inverted and rescaled) to quarterly GDP growth. Panel **b** panel gives daily values of the fever curve along with important policy decisions

Panel a of Fig. 1 shows the *f*-curve (on an inverted scale) jointly with real gross domestic product (GDP) growth: the indicator closely tracks economic crises. It presages the downturn during the Global Financial Crisis, responds to the removal of the minimum exchange rate and to the euro area debt crisis. The *f*-curve also responds strongly to the COVID-19 crisis (see panel b). The indicator starts to rise in late February. By then, it became evident that the COVID-19 crisis will hit most European countries; in Switzerland, the first large events were canceled. It reaches a peak shortly after the lockdown. Afterward, the fever curve gradually declines with news about economic stimulus packages and gradual loosening of the lockdown. The peak during the COVID-19 crisis is comparable with the Global Financial Crisis. But the speed of the downturn is considerably higher. In addition, so far, the crisis is less persistent. Up to June 4, 2020, the *f*-curve improved to 1/4 of its peak value during the lockdown.

The indicator has several advantages we hope will make it useful for policy makers and the public at large. The methodology of the *f*-curve is simple; the data selection process is based on economic theory and intuition; the data sources are publicly available, and we provide the program codes and daily updates on https://github.com/dankaufmann/f-curve/.^2^ Moreover, additional daily indicators that track economic activity are easily integrated in the modeling framework.

There are various initiatives in Switzerland and abroad to satisfy the demand for reliable high-frequency information during the COVID-19 crisis. Becerra et al. (2020) develop sentiment indicators using Internet search engine data. Brown and Fengler (2020) provide information on Swiss consumption behavior based on debit and credit card payment data. Eckert and Mikosch (2020) develops a daily mobility index using data on traffic, payments, and cash withdrawals. For the USA, economists at the Federal Reserve Bank of New York estimate a weekly index of economic activity based on retail sales, unemployment insurance claims, and other rapidly available data on production, prices, and employment (Lewis et al. 2020). Moreover, Buckman et al. (2020) create a daily news sentiment indicator that leads the US traditional consumer sentiment based on surveys. Our paper is the first, to the best of our knowledge, to combine daily information from newspapers and financial market data in a daily measure of economic activity for Switzerland.

In what follows, we describe the data and methodology. Then, we provide an analysis of the in- and out-of-sample performance. The last section concludes.

## Data

We use publicly available bond yields underlying the SIX Swiss Bond Indices ^*Ⓡ*^ (SIX 2020a). These data are available on a daily basis and with a delay of 1 day. Because many bond yields start only around 2007, we extend the series with a close match of government and corporate bond yields from the Swiss National Bank (see Table A.2 and Figure A.2 in the Online Appendix).^3^ Then, we compute various spreads that should be correlated with economic activity: a government bond term spread (8Y - 2Y), the interest rate differential vis-à-vis the euro area (1Y), and risk premia of short- and long-term corporate debt. Besides interest rate spreads for Switzerland, we compute risk premia of foreign companies that issue debt in Swiss franc for short- and long-term debt. We also include term spreads for the USA and for the euro area. For the latter, we use short-term interest rates in euro (European Central Bank 2020) and long-term yields of German government debt (Deutsche Bundesbank 2020). In addition, we include two implied volatility measures of the Swiss and US stock market. Swiss data stem from SIX (2020b) and are published with a delay of one day. The US data stem from the Chicago Board Options Exchange (2020).

These financial market data should be related to the Swiss business cycle. Stuart (2020) shows that the term spread exhibits a lead on the Swiss business cycle.^4^Kaufmann (2020) argues that a narrowing of the interest rate differential appreciates the Swiss franc and thereby dampens economic activity. Risk premia are correlated with the default risk of companies, which should increase during economic crises. Finally, recent research documents an increase in uncertainty during economic downturns (Baker et al. 2016; Scotti 2016). There are various ways to measure uncertainty (see e.g., Dibiasi and Iselin 2016). Because we aim to exploit quickly and freely available financial market data, we prefer a measure of stock market volatility.

We complement the financial market data with sentiment indicators based on Swiss newspapers. We extract headlines and lead texts from the online archives of the *Tages-Anzeiger*, the *Neue Zürcher Zeitung*, and the *Finanz und Wirtschaft*.^5^ We focus on the headline and lead text as these are publicly available and often contain the key messages of the articles. To reduce the number of potentially relevant articles, and to decompose the sentiment indicator into a domestic and foreign part, we only use articles satisfying specific search queries (see Table A.3 in the Online Appendix for a detailed description).

To calculate a news sentiment, we use the lexical methodology (see, e.g., Ardia et al. 2019; Shapiro et al. 2017; Thorsrud, 2020). First, we filter out irrelevant information.^6^ Second, we identify positive and negative words using the lexicon developed by Remus et al. (2010). Finally, we calculate for each article *n* and each day *t* a sentiment score:

$$S_{t,n} = \frac{\#P_{t,n} - \#N_{t,n}}{\#T_{t,n}} \ ,$$

where *#P*_*t*,*n*_,*#N*_*t*,*n*_,*#T*_*t*,*n*_ represent, for each article and each time period, the number of positive, negative, and total words, respectively. Finally, we compute a simple average over all articles to obtain daily indicators for articles about the domestic and foreign economy.

News sentiment indicators receive more and more attention for forecasting economic activity. Buckman et al. (2020) show that during the COVID-19 pandemic, news sentiment indicators provide reliable and early information on the economy, even compared to quickly available survey data. Moreover, Ardia et al. (2019) show that news sentiment helps forecast the US industrial production growth.

## Methodology

The financial market data and news indicators are quite volatile, but also they are correlated with each other. To parsimoniously summarize the information content of the data and remove idiosyncratic noise, we estimate a factor model in static form:^7^

$$X = F\Lambda + e$$

The model comprises *N* variables and *T* daily observations. Therefore, the data matrix *X* is (*T*×*N*), the common factors *F* are (*T*×*r*), the factor loadings *Λ* are (*r*×*N*), and the unexplained error term *e* is (*T*×*N*). The advantage of a factor model is that we can parsimoniously summarize the information content in the large data matrix *X* with a relatively small number of common factors *r*. Assuming that the idiosyncratic components are only weakly serially and cross-sectionally correlated, we can estimate the factors and loadings by principal components (Bai and Ng 2013; Stock and Watson 2002).^8^ Our main indicator is the first principal component of the static factor model. We normalize the indicator that it increases during crises.^9^

Because this factor has no clear economic interpretation, we decompose it into a contribution from domestic and foreign fluctuations. Suppose that there are only two factors driving the variables. One factor captures foreign fluctuations. The other factor captures domestic fluctuations. We allow for spillovers from abroad to the domestic economy, but not vice versa. Under these assumptions, the factor model reads:

$$\left[\begin{array}{cc} X & X^{*} \end{array}\right] = \left[\begin{array}{cc} f & f^{*}\end{array}\right] \left[\begin{array}{cc} \lambda_{11} & 0 \\ \lambda_{21} & \lambda_{22} \end{array}\right] + e $$

where *X*,*X*^∗^ denote the data matrices comprising domestic and foreign variables, respectively. In addition *f*,*f*^∗^ represent the domestic and foreign factors and *λ*_11_,*λ*_21_,*λ*_22_ are the loading matrices.

To estimate this factor model, we can use an iterative procedure inspired by Boivin et al. (2009). First, we estimate the foreign factor only on foreign data. This imposes that foreign variables only load on the foreign factor. Second, we estimate the domestic factor on $\tilde X$, where

$$\tilde X = X - \lambda_{21}f^{*} \ ,$$

removes variation explained by the foreign factor. We can estimate *λ*_21_ for every indicator comprised in *X* in a regression on the domestic and foreign factor. Because this regression depends on the value of the domestic factor, we repeat this step 50 times (see Boivin et al., 2009; Kaufmann and Lein 2013, for more details). Finally, we can estimate a decomposition by regressing the *f*-curve on the domestic and foreign factors. This procedure does not guarantee that the decomposition adds up exactly to the overall factor. However, the unexplained rest turns out to be relatively small. The decomposition involves additional estimation steps that may reduce the forecast accuracy; therefore, we only use this decomposition for the in-sample interpretation, but not for out-of-sample forecasting.

## Analysis

The *f*-curve should primarily be used to quickly detect turning points of the business cycle. As such, it is correlated or leading many key macroeconomic variables (see Figure A.4 in the Online Appendix). In its current form, we have not optimized the indicator to track any particular measure of economic activity. We therefore first focus on the in-sample information content of the *f*-curve, highlighting that it is available earlier than most other leading indicators. For the sake of illustration, however, we additionally provide an evaluation of its pseudo out-of-sample performance for forecasting real GDP growth.

### In-sample analysis

**Fig. 2:**
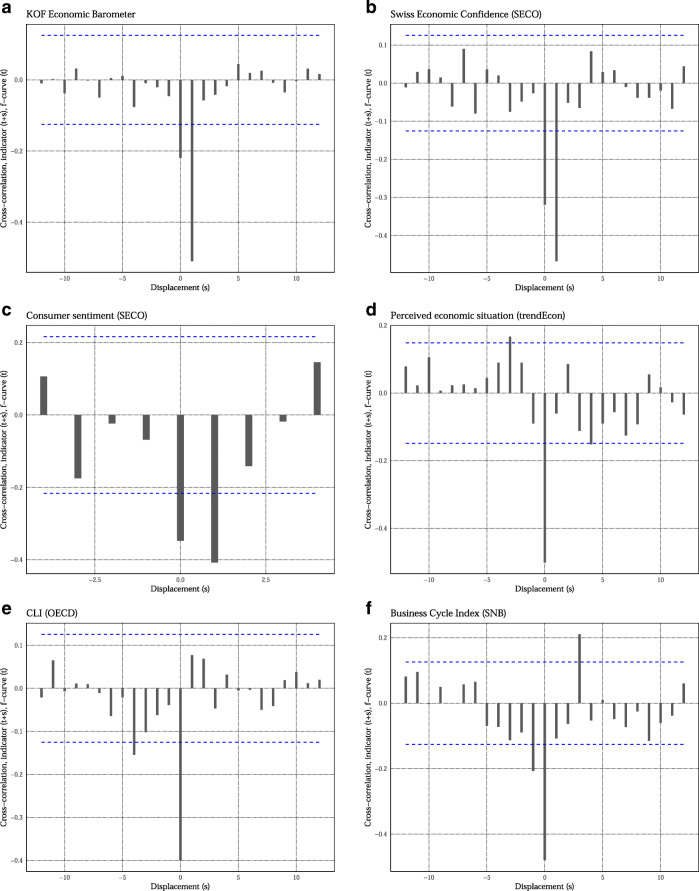
Cross-correlation with other indicators. Cross-correlation between the *f*-curve and other prominent leading and sentiment indicators. We aggregate all data either to quarterly frequency (consumer sentiment) or monthly frequency (remaining indicators). The dashed lines give 95% confidence intervals. A bar outside of the interval suggests a statistically significant correlation between the indicators at a lead/lag of *s*. Before computing the cross-correlation, the series have been pre-whitened with an AR(p) model (see Neusser 2016, Ch. 12.1). The lag order has been determined using the Bayesian information criterion. The only exception is the OECD CLI for which we used an AR(4) model

To compare the in-sample information content of the *f*-curve to other leading indicators, we perform a cross-correlation test (see Neusser, 2016, Ch. 12.1).^10^ Figure 2 shows a substantial correlation between the *f*-curve and many prominent leading indicators.^11^ There is a coincident or leading relationship with the KOF Economic Barometer, SECO’s Swiss Economic Confidence, the Organisation for Economic Co-operation and Development composite leading indicator (OECD CLI), and consumer confidence.^12^ There is a coincident relationship with trendEcon’s perceived economic situation. This daily indicator starts only in 2006, however. There is a significant lagging relationship with the SNB’s Business Cycle Index. But this index is published with a relevant delay. Overall, these results suggest the *f*-curve provides sensible information comparable with other existing indicators. The key advantage of the *f*-curve is its prompt availability and that it is available on a longer time period.

**Fig. 3:**
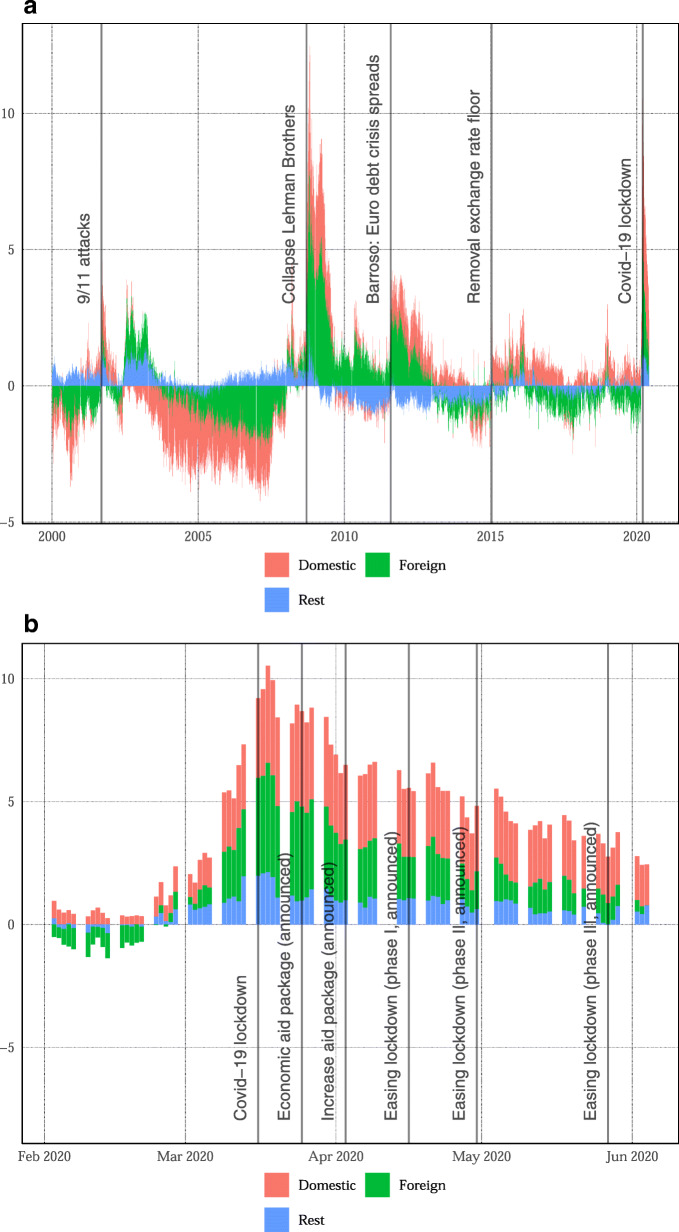
Decomposition domestic and foreign variables. Decomposition of the *f*-curve into foreign factors, domestic factors, and an unexplained rest

Another advantage is that we can decompose its fluctuations into domestic and foreign factors. Panel a of Fig. 3 shows that the foreign contribution rises after the collapse of Lehman Brothers, but also, during the euro area debt crisis. By contrast, the domestic contribution rises after the removal of the minimum exchange rate in 2015, but also, during the COVID-19 crisis. Focusing on the COVID-19 crisis, panel b shows the indicator rose already in the last week of February, before the actual COVID-19 lockdown. It reaches a peak during the first week of the lockdown and gradually declines thereafter. About half of the increase in the indicator can be traced back to foreign developments. Although the domestic lockdown is important, the *f*-curve suggests the Swiss economy would have suffered even in the absence of these restrictions. During the last 4 weeks, the contribution from foreign variables declines. The domestic contribution, however, remains elevated. Therefore, while the negative foreign demand shock seems to become less important, the model suggests economic activity will remain subdued also due to domestic headwinds.

### Pseudo out-of-sample evaluation

How reliable is the *f*-curve? To answer this question, we perform a pseudo-real-time forecast evaluation. Therefore, we use the real-time data set for quarterly GDP vintages by Indergand and Leist (2014).^13^ In the evaluation, we use the following direct forecasting model:

$$y_{\tau+h} = \alpha_{h} + \beta_{h,1}f_{\tau|t} + \beta_{h,2}f_{\tau-1}+\nu_{\tau+h}$$

where *y*_*τ*_ denotes quarterly GDP growth, *h* is the forecast horizon, *τ* gives time in quarterly frequency, and *t* denotes time in daily frequency. *f*_*τ*|*t*_ is our best guess of the *f*-curve for the entire quarter based on daily information at time *t*. We compute *f*_*τ*|*t*_ and *f*_*τ*_ as the simple average of available daily observations for a given quarter. Finally, *ν*_*τ*+*h*_ is an error term. At the time of our last update, *τ*= 2020 Q2 and *t*= 4 June 2020.

We then conduct a forecast based on the state of information when a new quarterly GDP vintage is published by SECO.^14^ This yields 70 nowcasts (69 one-quarter-ahead forecasts). These forecasts are compared to three benchmarks. First, we compare the forecasts to the first quarterly release of GDP growth for the corresponding quarter. Because quarterly GDP is substantially revised ex-post, we treat the initial quarterly GDP release as a forecast of the true GDP figure. Second, we use an autoregressive model of order 1, AR(1), estimated on the corresponding real-time vintage for GDP growth. Third, using the same forecasting equation as for the *f*-curve, we forecast GDP growth using the KOF Economic Barometer, a prominent monthly composite leading indicator (Abberger et al. 2014). To compute the forecast errors, we use the last available release of quarterly GDP from June 3, 2020.

Table 1 panel a shows the root-mean-squared error (RMSE) of the *f*-curve is higher than the one of the first official GDP release. However, the difference is not statistically significant. The advantage of the *f*-curve is, of course, that its value for the entire quarter is available about 2 months earlier than the first GDP release. In addition, we compare the *f*-curve to an AR(1) model. Panel b shows we outperform the AR(1) benchmark. The RMSE is 18% lower for the current quarter. Moreover, the difference in forecast accuracy is statistically significant. For the next quarter, however, the *f*-curve does not provide a more accurate forecast than the AR(1) model. Panel c shows that the *f*-curve yields similar results as the KOF Economic Barometer. The difference in the RMSE is never statistically significant. This suggest the advantage of our indicator primarily lies in its prompt availability.

**Table 1:** Pseudo-real-time evaluation. Root-mean-squared errors (RMSE) for forecasts on days with a new quarterly GDP release. A lower RMSE implies higher predictive accuracy. *h*=0 (*h*=1) denotes the forecast for the current (next) quarter. We use three benchmarks. First, we use the first quarterly release of the corresponding quarter (panel a). Second, we use an AR(1) model (panel b). Third, we use the KOF Economic Barometer (panel c). The Diebold-Mariano-West (DMW) test provides a *p* value for the null hypothesis of equal predictive accuracy against the alternative written in the column header (Diebold and Mariano 2002; West 1996). We assume a quadratic loss function

(a) Real GDP growth: First release vs. *f*-curve				
	RMSE	RMSE	Relative RMSE	DMW test (*p* value)
	First release	*f*-curve	First release/*f*-curve	First release <*f*-curve
*h*=0	0.45	0.57	0.79	0.177
*h*=1	0.45	0.7	0.64	0.042
(b) Real GDP growth: *f*-curve vs. AR(1)				
	RMSE	RMSE	Relative RMSE	DMW test (*p* value)
	*f*-curve	AR(1)	*f*-curve/AR(1)	*f*-curve < AR(1)
*h*=0	0.57	0.7	0.82	0.039
*h*=1	0.7	0.7	0.99	0.458
(c) Real GDP growth: *f*-curve vs. KOF Economic Barometer				
	RMSE	RMSE	Relative RMSE	DMW test (*p* value)
	*f*-curve	Barometer	*f*-curve/Barometer	*f*-curve < Barometer
*h*=0	0.57	0.6	0.95	0.162
*h*=1	0.7	0.65	1.07	0.832

We perform a subsample analysis in Table 2. The current vintage of GDP, which we use to compute the forecast errors, will likely be revised in the future. One of the reasons is that future vintages will include annual GDP estimates by the SFSO, which are based on comprehensive firm surveys. Therefore, we restrict the sample to years where the GDP figures already include these annual figures (panel a). The *f*-curve performs better on this sample. In fact, the RMSE is almost identical to the RMSE of the first GDP release for the current quarter. A similar picture emerges when excluding economic crises (panel b). This implies that the *f*-curve does not only signal deep economic crises, but tracks the economy well also during normal times.

**Table 2:** Subsample evaluation for real GDP growth: First release vs. *f*-curve. Root-mean-squared errors (RMSE) for forecasts on days with a new quarterly GDP release. A lower RMSE implies higher predictive accuracy. *h*=0 (*h*=1) denotes the forecast for the current (next) quarter. Panel (a) shows the evaluation for GDP figures that include the annual SFSO estimates (until 2018). Panel (b) excludes economic crises. As benchmark, we use the first quarterly release of the corresponding quarter. The Diebold-Mariano-West (DMW) test provides a *p* value for the null hypothesis of equal predictive accuracy against the alternative written in the column header (Diebold and Mariano 2002; West 1996). We assume a quadratic loss function

(a) Only when annual GDP estimates available (2000–2018)				
	RMSE	RMSE	Relative RMSE	DMW test (*p* value)
	First release	*f*-curve	First release/*f*-curve	First release <*f*-curve
*h*=0	0.47	0.46	1.03	0.605
*h*=1	0.46	0.6	0.77	0.061
(b) Without crises (excluding 2008, 2009, 2020)				
	RMSE	RMSE	Relative RMSE	DMW test (*p* value)
	First release	*f*-curve	First release/*f*-curve	First release <*f*-curve
*h*=0	0.4	0.4	1	0.504
*h*=1	0.4	0.43	0.91	0.166

**Fig. 4:**
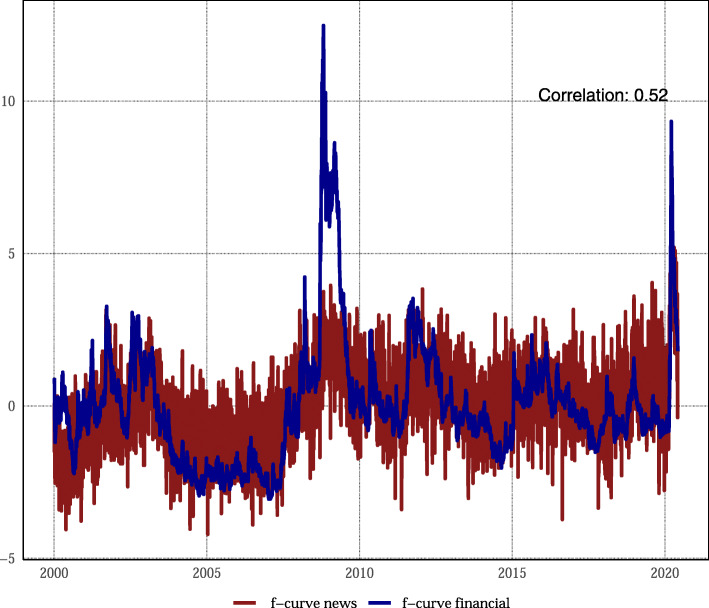
Comparison news and financial market data. Two indicators estimated only on financial market and news data, respectively

Are the financial market or news data more important for the forecasting performance of the *f*-curve? Figure 4 shows two indicators only calculated with financial market and news data, respectively. Although the indicators are positively correlated, there are two key differences. First, the financial market data respond more strongly during crises. Second, the news data are more volatile.^15^ This suggests the financial market data provide a more accurate signal of the business cycle than the news data. Table 3 confirms this view. The RMSE for an indicator based only on financial market variables amounts to 0.57, the same as for the overall *f*-curve. Meanwhile, the RMSE of a forecast based only on news data amounts to 0.64. The news data does not worsen the *f*-curve because the factor model including financial market data removes the idiosyncratic fluctuations; taken in isolation, however, the news indicator performs worse.

**Table 3:** Comparison news vs. financial data. Root-mean-squared errors (RMSE) for forecasts on days with a new quarterly GDP release. A lower RMSE implies higher predictive accuracy. *h*=0 (*h*=1) denotes the forecast for the current (next) quarter. Panel (a) shows the evaluation for an indicator based only on financial market data. Panel (b) shows the evaluation for an indicator based only on news data. As benchmark, we use the first quarterly GDP release for the corresponding quarter. The Diebold-Mariano-West (DMW) test provides a *p* value for the null hypothesis of equal predictive accuracy against the alternative written in the column header (Diebold and Mariano 2002; West 1996). We assume a quadratic loss function

(a) Only financial market data				
	RMSE	RMSE	Relative RMSE	DMW test (*p* value)
	First release	*f*-curve	First release/*f*-curve	First release <*f*-curve
*h*=0	0.45	0.57	0.79	0.19
*h*=1	0.45	0.71	0.63	0.042
(b) Only news data				
	RMSE	RMSE	Relative RMSE	DMW test (*p* value)
	First release	*f*-curve	First release/*f*-curve	First release <*f*-curve
*h*=0	0.45	0.64	0.71	0.047
*h*=1	0.45	0.7	0.63	0.019

**Fig. 5:**
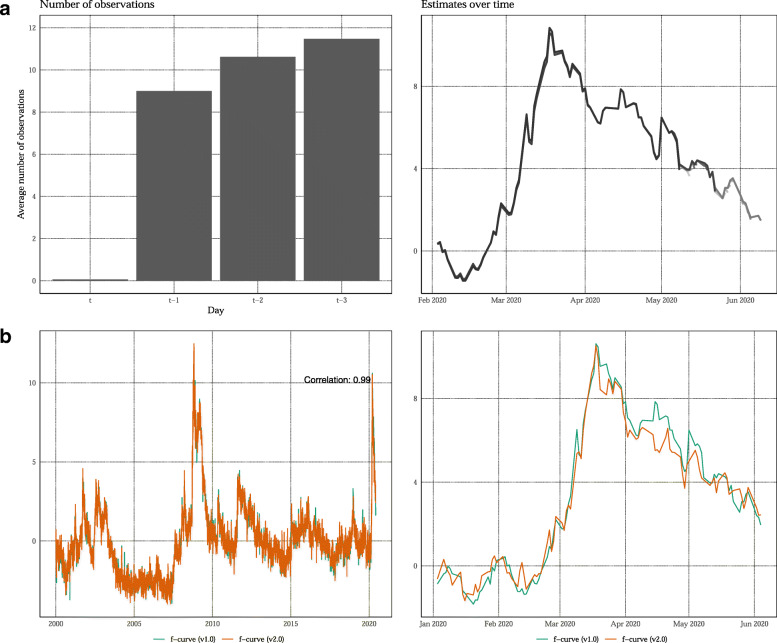
Real-time results since initial version of the *f*-curve. Panel **a**: average number of observations available for calculation the *f*-curve (left figure). The different shades of gray represent estimates over time from May 11, 2020, to May 29, 2020 (right figure). Panel **b**: Estimates of the *f*-curve using the methodology in the Working Paper (v1.0) and the current version (v2.0)

Although it is too early to judge the actual real-time performance of the indicator, Fig. 5 provides some preliminary results on the stability of the *f*-curve over time. One reason why the indicator is revised is that not all data series are available in real-time (ragged edge problem). Panel (a) shows results over the first month we updated the indicator on a daily basis. On average, more than 8 out of 12 series are available with a delay of 1 day. After 3 days, almost all indicators are available.

The main reason why the average lies below 12 is that the archive of *Tages-Anzeiger* has not been updated since 12 May 12, 2020.^16^ Therefore, we augmented the indicator with information from this newspapers’ online edition. Adding this source resulted in a slightly larger revision of the indicator compared to the Working Paper version (see panel b). However, the correlation between the old and new version is 0.99 and the broad picture during the COVID-19 crisis is identical.

## Concluding remarks

We develop a daily indicator of Swiss economic activity. A major strength of the indicator is that it can be updated with a delay of only 1 day. An evaluation of the indicator shows that it is not only correlated with other business cycle indicators but also accurately tracks Swiss GDP growth. Therefore, the *f*-curve provides an accurate and flexible framework to track Swiss economic activity at high frequency.

Having said that, there is still room for improvement. We see six promising avenues for future research. First, the news sentiment indicators could exploit other publicly available news sources, in particular, newspapers from the French- and Italian-speaking parts of Switzerland. Second, we could use a topic modeling algorithm, instead of our own search queries, to classify news according to countries, sectors, and economic concepts (see e.g., Thorsrud, 2020). Third, the lexicon could be tailored specifically to economic news (see e.g., Shapiro et al., 2017). Fourth, we could examine the predictive ability of multiple factors and for other macroeconomic data. Fifth, the information could be used to disaggregate quarterly GDP and industrial production into monthly or even weekly series. Finally, it would be desirable to collect and exploit the information from many different daily indicators that are currently developed into one single composite indicator or indicator data set. Exploiting all this new information will likely further improve our understanding of health of the Swiss economy at high frequency.

## Supplementary information

**Additional file 1** The Online Appendix to this paper is available on https://www.dankaufmann.com/publications/.

**Additional file 2** Replication files. Codes for replication of the main indicator are available on https://github.com/dankaufmann/f-curve/.

## Data Availability

Data are available on https://github.com/dankaufmann/f-curve/.

## Abbreviations

AR(p): Autoregressive model of order *p*
CH: Switzerland
CLI: Composite leading indicator
COVID-19: Coronavirus disease of 2019
EUR: Europe /
*f*-curve: Fever curve
FuW: *Finanz und Wirtschaft*
GDP: Gross domestic product
HTML: HyperText Markup Language
ILO: International Labor Organization
KOF: *Konjunkturforschungsstelle*
NZZ: *Neue Zürcher Zeitung*
OECD: Organisation for Economic Co-operation and Development
Q: Quarter
RMSE: Root-mean-squared error
SECO: State Secretariat for Economic Affairs
SFSO: Swiss Federal Statistical Office
SNB: Swiss National Bank
TA: *Tages-Anzeiger*
Y: Year

## Footnotes

1 See Table A.1 in the Online Appendix for publication lags of some important macroeconomic data and leading indicators.

2 We plan to continuously extend the indicator. We therefore welcome suggestions for improvements and extensions.

3 Data from the Swiss National Bank are published with a longer delay. Therefore, these bond yields cannot be used to track the economy on a daily basis.

4 We therefore move forward all term spreads by half a year.

5 During the first month of daily updates, we noticed that the *Tages-Anzeiger* updates its archive with a relevant delay or not at all. Therefore, in the revised version of the indicator, we additionally include articles from the *Tages-Anzeiger* website.

6 We remove HyperText Markup Language (HTML) tags, punctuation, numbers, and so-called stop words (e.g., the German words *der*, *wie*, *ob*). The stop words are provided by Feinerer and Hornik (2019). Also, we transform all letters to lowercase.

7 The news indicators are much more volatile than the financial market data (see Figure A.1 in the Online Appendix). We therefore compute a one-sided 2-day moving average before including them in the factor model.

8 To account for missing values, we compute the indicator only if at least five underlying data series are observed. Moreover, we remove all weekends. Then, we interpolate few additional missing values using an EM-algorithm (Stock and Watson 2002), after normalizing the data to have zero mean and unit variance. For interpolation, we choose a relatively large number of factors for interpolating the data (*r*=4). Finally, we estimate the *f*–curve as the first principal component of the interpolated data set.

9 An interesting extension would be to examine whether more than one factor comprises relevant information for Swiss economic activity. We leave this extension for future research.

10 It is noteworthy that other indicators are estimated or smoothed such that they undergo substantial revisions over time; moreover, some of the indicators are published with significant delays (see Table A.1 in the Online Appendix); finally, some are based on lagged data (see, e.g., OECD 2010).

11 Figure A.3 in the Online Appendix provides plots of these indicators.

12 All data sources are given in the Online Appendix.

13 The evaluation is not strictly a real-time forecast evaluation because we use three types of in-sample information. First, the *f*-curve is constructed based on knowledge of the business cycle in the past, in particular, the Global Financial Crisis. Second, the link of the underlying indicators with new data is based on inspecting whether different data sources are highly correlated. Third, the normalization of the indicators in the factor model may introduce revisions that we do not account for in the forecast evaluation. Arguably, using this in-sample information in the evaluation makes sense if the goal of the evaluation is to show whether the indicator is useful going forward rather than whether the indicator would have been useful in the past.

14 These dates stem from Indergand and Leist (2014).

15 This is also because we smooth the news indicator with a moving-average of only 2 days. Comparable studies smooth over a longer time period. For example, Thorsrud (2020) uses a moving average of 60 days. On the one hand, this reduces the volatility of the news sentiment. On the other hand, this obviously renders the indicator less useful for detecting rapid daily changes.

16 On rare occasions, the websites of other sources were not available.
