# Supplementary material for: A daily fever curve for the Swiss economy
Source: Swiss J Econ Stat. 2020 Jul 9;156(1):6. doi: 10.1186/s41937-020-00051-z (PMC7347051; doi:10.1186/s41937-020-00051-z)
Supplement: Supplementary file 1 — Additional file 1 The Online Appendix to this paper is available on https://www.dankaufmann.com/publications/. [file 41937_2020_51_MOESM1_ESM.pdf]

# A daily fever curve for the Swiss economy

Marc Burri*    Daniel Kaufmann† ‡§

12 June 2020

## A Online Appendix (not for publication)

Table A.1 — Macroeconomic data and leading indicators

| | Type | Publication | Frequency | Source | Comments |
|---|---|---|---|---|---|
| GDP | Target | +9 weeks | Quarter | SECO | First publication subject to further revisions |
| Employment | Target | +9 weeks | Quarter | SFSO | |
| Registered unemployment | Target | +1 week | Month | SECO | |
| ILO unemployment | Target | +6 weeks | Month | SFSO | |
| Output gap | Target | $>$ +4 months | Quarter | SNB | |
| SNB Business Cycle Index | Indicator | $>$ +2 months | Month | SNB | |
| Internet search sentiment | Indicator | +1 day | Day | trendEcon | Indicator based on internet search engine |
| KOF Economic Barometer | Indicator | +0 days | Month | KOF | Some underlying data probably missing at the end of the sample |
| Consumer sentiment | Indicator | +4 weeks | Quarter | SECO | Survey during first month of quarter. Indicator published at beginning of second month |
| OECD CLI | Indicator | $>$ +1 week | Month | OECD | Many underlying data are lagged two months |

*Notes:* Publication lags between the last day of the variable frequency (i.e. last day of the quarter or last day of the month) and the publication date of a recent release. Therefore, all publication lags are approximate and may change over time.

*marc.burri@unine.ch, University of Neuchtel, Institute of Economic Research, Rue A.-L. Breguet 2, CH-2000 Neuchâtel,
†daniel.kaufmann@unine.ch, University of Neuchâtel, Institute of Economic Research, Rue A.-L. Breguet 2, CH-2000 Neuchâtel,
‡KOF Swiss Economic Institute
§Corresponding author

Table A.2 — Data underlying *f*–curve

| | Type | Publication | Frequency | Source | Comments |
|---|---|---|---|---|---|
| Term spread CH | *f*–curve | +1 day | Day | SIX, SNB | 8Y − 2Y. SNB data used before SIX data available. Maturity of SIX data is approximate |
| Term spread USA | *f*–curve | +1 day | Day | Fed Board | 10Y − 2Y |
| Term spread Europe | *f*–curve | +1 day | Day | Buba, ECB | 10Y Germany − 1Y euro area. 1Y EUR Libor used before 2004 |
| Risk premium CH | *f*–curve | +1 day | Day | SIX, SNB | 8Y AAA-AA − 8Y government. SNB data for debt issues by banks used before SIX data available. Maturity of SIX data is approximate |
| Short-term risk premium CH | *f*–curve | +1 day | Day | SIX | 1-3Y AAA-BBB − 1-3Y government. Start in 2008 |
| Risk premium foreign | *f*–curve | +1 day | Day | SIX, SNB | 8Y Foreign corp. − 8Y government. SNB data used before SIX data available. Average of various credit ratings |
| Short-term risk premium foreign | *f*–curve | +1 day | Day | SIX | 1-3Y AAA-AA − 8Y government. Start in 2008 |
| Stock market volatility CH | *f*–curve | +1 day | Day | SIX | |
| Stock market volatility USA | *f*–curve | +1 day | Day | CBOE | |
| Interest rate differential | *f*–curve | +1 day | Day | SIX, SNB, ECB | 1-3 year government bonds CH − 1 year government bond yields euro area. 1Y EUR Libor used before 2004 |
| Domestic news sentiment | *f*–curve | +1 day | Day | FuW, NZZ, TA | More details in Table A.3 |
| Foreign news sentiment | *f*–curve | +1 day | Day | FuW, NZZ, TA | More details in Table A.3 |

*Notes:* The SIX Swiss Exchange AG disclaimer applies to the SIX data: https://www.six-group.com/exchanges/download/market/data_services/six

Table A.3 — Queries underlying news indicators

| | URL | Keywords |
|---|---|---|
| | **Domestic news sentiment** | |
| FuW | fuw.ch/unternehmen/<br>fuw.ch/makro/ | We use all articles listed in *Makro* and *Unternehmen* and select those containing the word *schweiz** in either lead text, tag or cateogry. |
| NZZ | zeitungsarchiv.nzz.ch | [*konjunktur** OR *wirtschaft** OR *rezession**] AND *schweiz** |
| TA | tagesanzeiger.ch/zeitungsarchiv-930530868737 | [*konjunktur* OR *wirtschaft* OR *rezession*] AND *schweiz* |
| TA Web | tagesanzeiger.ch | [*konjunktur* OR *wirtschaft* OR *rezession*] AND *schweiz* |
| | **Foreign news sentiment** | |
| FuW | fuw.ch/unternehmen/<br>fuw.ch/makro/ | We use all articles listed in *Makro* and *Unternehmen* and select those containing [*ausland* OR *eu* OR *euro** OR *deutsch** OR *us** OR *amerika**] in either lead text, tag or cateogry. |
| NZZ | zeitungsarchiv.nzz.ch | [*konjunktur** OR *wirtschaft** OR *rezession**] AND [*ausland* OR *eu* OR *euro** OR *deutsch** OR *us** OR *amerika**] |
| TA | tagesanzeiger.ch/zeitungsarchiv-930530868737 | [*konjunktur* OR *wirtschaft* OR *rezession*] AND [*ausland* OR *eu* OR *euro* OR *europa* OR *deutschland* OR *us* OR *usa* OR *amerika*] |
| TA Web | tagesanzeiger.ch | [*konjunktur* OR *wirtschaft* OR *rezession*] AND [*ausland* OR *eu* OR *euro* OR *europa* OR *deutschland* OR *us* OR *usa* OR *amerika*] |

*Notes:* Since the *Finanz und Wirtschaft* is a business newspaper, we do not restrict the search with keywords related to the economy. The asterisk (*) represents a wildcard search operator. E.g. the query *schweiz** matches also *schweizerische*. Wildcards are allowed only in the NZZ archive.

Figure A.1 — Daily indicators for $f$–curve

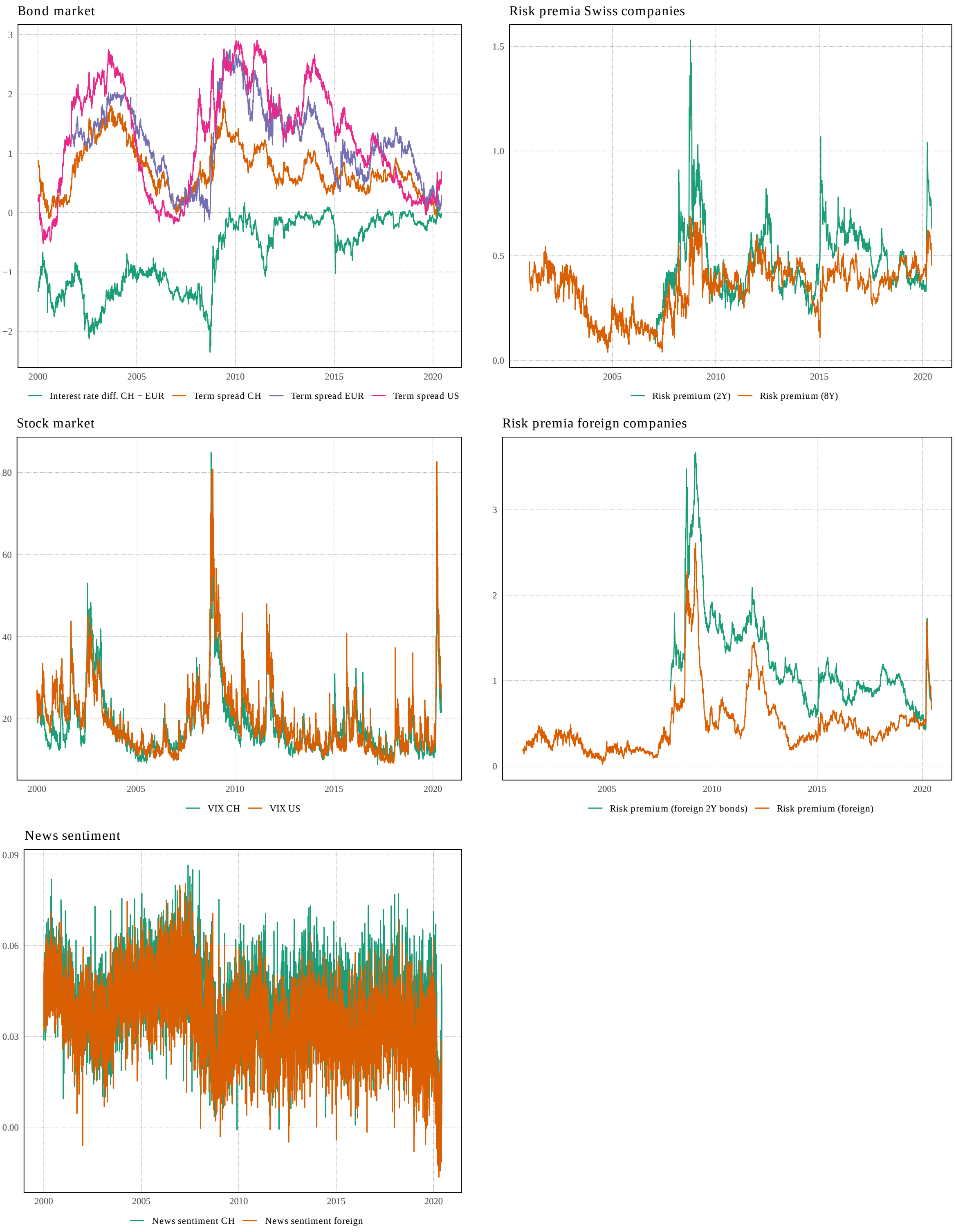

## Figure A.2 — Spliced data underlying *f*–curve

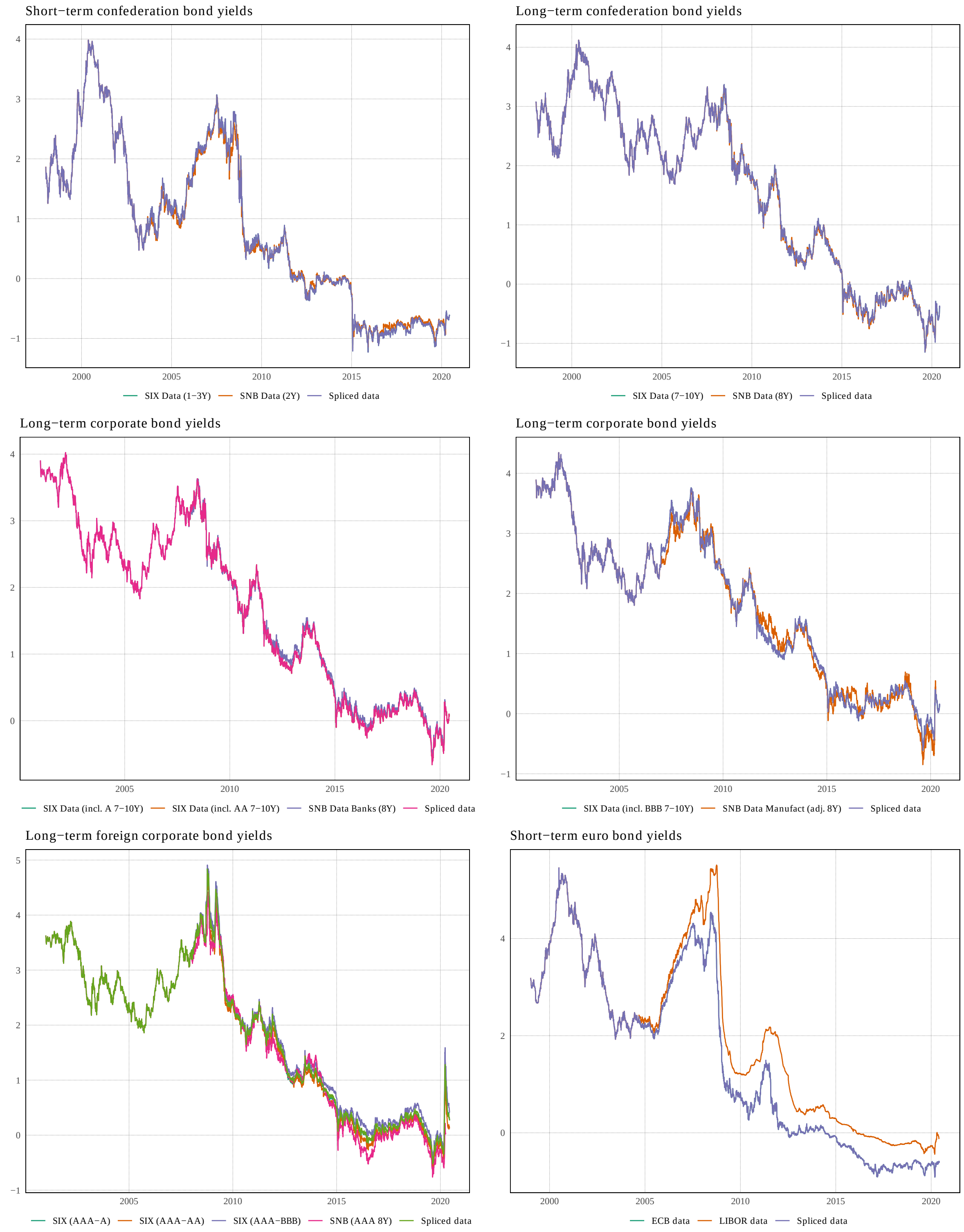

# Figure A.3 — Comparison with other indicators

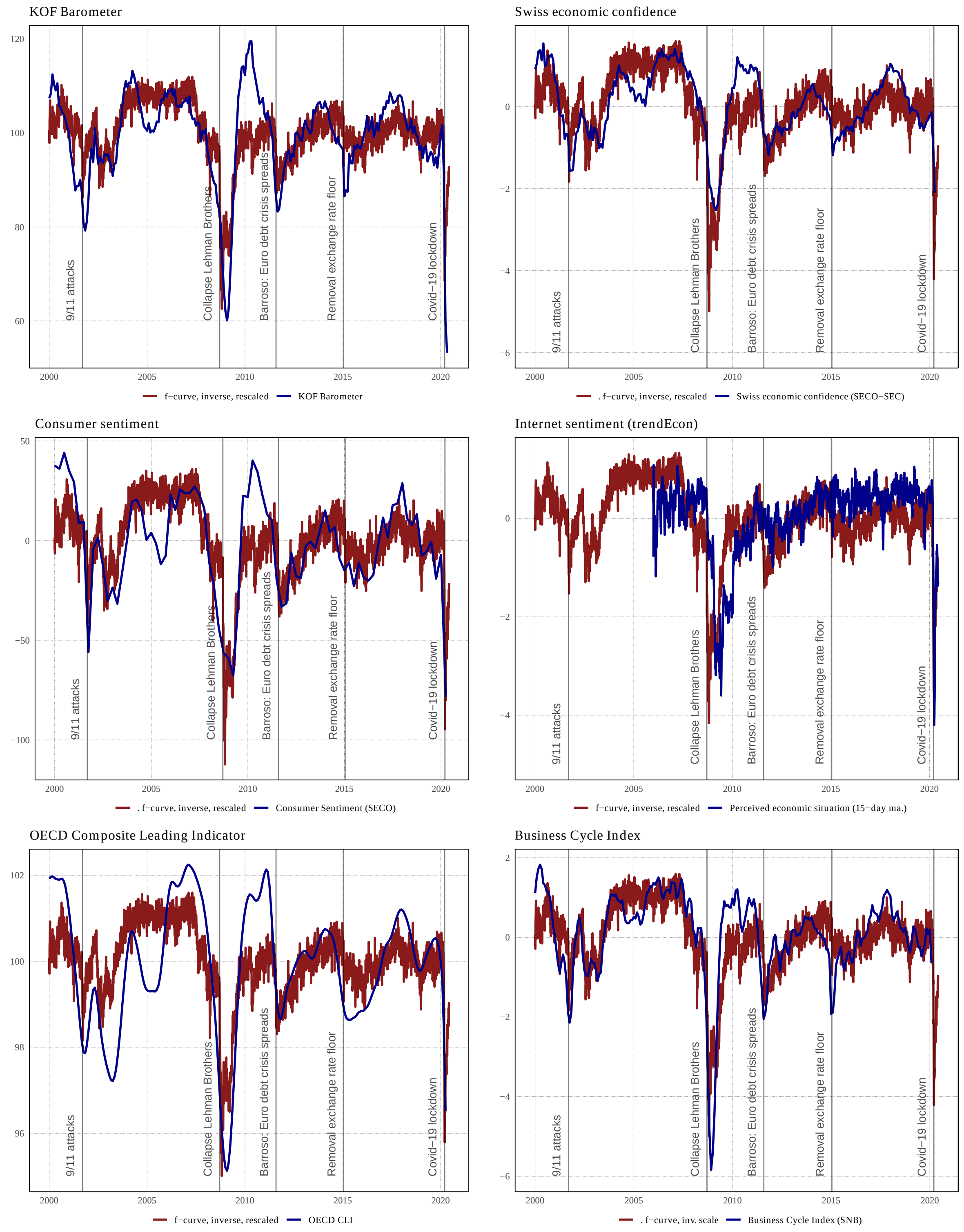


*Notes: f*–curve rescaled such that it roughly matches the mean and volatility of the other data series.

## Figure A.4 — Comparison with other macroeconomic data

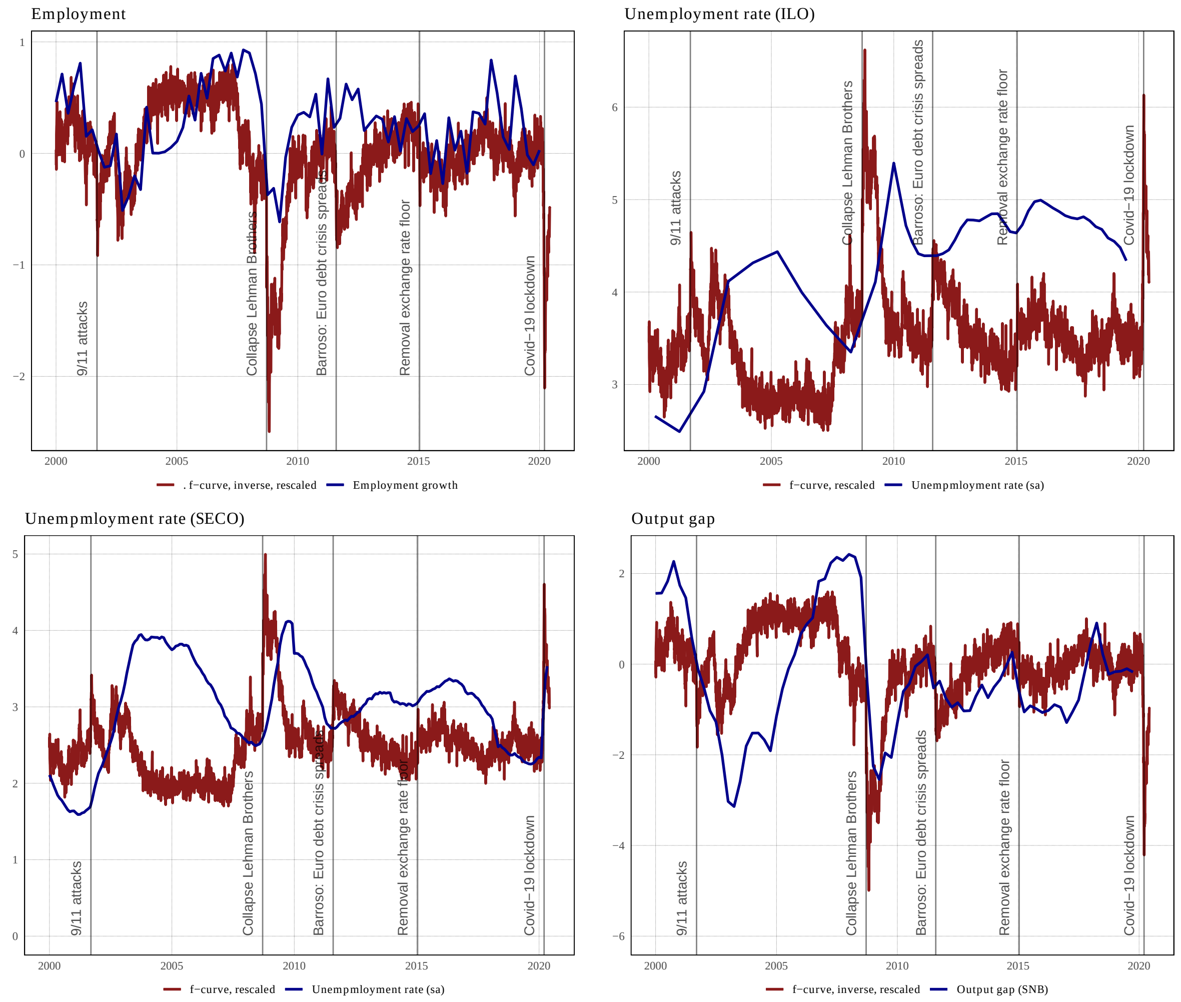


*Notes:* $f$–curve rescaled such that it roughly matches the mean and volatility of the other data series.
