# Supplementary material for: A daily fever curve for the Swiss economy
Source: Swiss J Econ Stat. 2020 Jul 9;156(1):6. doi: 10.1186/s41937-020-00051-z (PMC7347051; doi:10.1186/s41937-020-00051-z)
Supplement: Supplementary file 2 — Additional file 2 Replication files. Codes for replication of the main indicator are available on https://github.com/dankaufmann/f-curve/. [file 41937_2020_51_MOESM2_ESM.pdf]

RESEARCH

A daily fever curve for the Swiss economy

Marc Burri[1†] and Daniel Kaufmann[1,2*]

*Correspondence:
daniel.kaufmann@unine.ch
[1]Institute of Economic Research, University of Neuchâtel, Rue A.-L. Breguet 2, 2000 Neuchâtel, Switzerland
Full list of author information is available at the end of the article
†Equal contributor

Abstract

Because macroeconomic data is published with a substantial delay, assessing the health of the economy during the rapidly evolving Covid–19 crisis is challenging. We develop a fever curve for the Swiss economy using publicly available daily financial market and news data. The indicator can be computed with a delay of one day. Moreover, it is highly correlated with macroeconomic data and survey indicators of Swiss economic activity. Therefore, it provides timely and reliable warning signals if the health of the economy takes a turn for the worse.

Keywords: Covid–19; composite leading indicator; financial market data; news sentiment; Switzerland

Introduction

Because macroeconomic data is published with a substantial delay, assessing the health of the economy during the rapidly evolving coronavirus disease of 2019 (Covid–19) crisis is challenging. Usually, policy makers and researchers rely on early information from surveys and financial markets to construct leading indicators and estimate forecasting models (see, e.g., Abberger et al., 2014; Galli, 2018; Kaufmann and Scheufele, 2017; OECD, 2010; Stuart, 2020; Wegmüller and Glocker, 2019, for Swiss applications). These indicators and forecasts are published with a delay of one to two months.[1] During the Covid–19 crisis, however, we need high-frequency information to assess how stricter or looser health restrictions and economic stimulus programs affect the economy.

We propose a novel daily fever curve (f–curve) for the health of the Swiss economy based on publicly available financial market and news data. We construct risk premia on corporate bonds, term spreads, as well as stock market volatility indices starting in 2000. In addition, we collect short economic news from online newspaper archives. We then estimate a composite indicator which has the interpretation of a fever curve: As for monitoring the condition of a patient, an increase of the fever curve provides a reliable and timely warning signal if health takes a turn for the worse.

Panel (a) of Figure 1 shows the f–curve (on an inverted scale) jointly with real gross domestic product (GDP) growth: the indicator closely tracks economic crises. It presages the downturn during the Global Financial Crisis, responds to the removal of the minimum exchange rate, as well as to the euro area debt crisis. The f–curve also responds strongly to the Covid–19 crisis (see panel b). The indicator starts to rise in late February. By then, it became evident that the Covid–19 crisis

[1]See Table A.1 in the Online Appendix for publication lags of some important macroeconomic data and leading indicators.

Figure 1 A fever curve for the Swiss economy. Panel (a) compares the fever curve (inverted and rescaled) to quarterly GDP growth. Panel (b) panel gives daily values of the fever curve along with important policy decisions.

(a) Correlation with real GDP growth

(b) Evolution during the Covid–19 crisis

will hit most European countries; in Switzerland, the first large events were canceled. It reaches a peak shortly after the lockdown. Afterward, the fever curve gradually declines with news about economic stimulus packages and gradual loosening of the lockdown. The peak during the Covid–19 crisis is comparable with the Global Financial Crisis. But the speed of the downturn is considerably higher. In addition, so far the crisis is less persistent. Up to 4 June 2020, the f–curve improved to 1/4 of its peak value during the lockdown.

The indicator has several advantages we hope will make it useful for policy makers and the public at large. The methodology of the f–curve is simple; the data selection process is based on economic theory and intuition; the data sources are publicly available and we provide the program codes and daily updates on github.com/dankaufmann/f-curve/.[2] Finally, additional daily indicators that track economic activity are easily integrated in the modeling framework.

There are various initiatives in Switzerland and abroad to satisfy the demand for reliable high-frequency information during the Covid–19 crisis. Becerra et al. (2020) develop sentiment indicators using internet search engine data. Brown and Fengler (2020) provide information on Swiss consumption behavior based on debit and credit card payment data. Eckert (2020) develops a daily mobility index using data on traffic, payments, and cash withdrawals. For the U.S., economists at the Federal Reserve Bank of New Work estimate a weekly index of economic activity based on retail sales, unemployment insurance claims, as well as other rapidly available data on production, prices and employment (Lewis et al., 2020). Moreover, Buckman et al. (2020) create a daily news sentiment indicator that leads U.S. traditional consumer sentiment based on surveys. Our paper is the first, to the best of our knowledge, to combine daily information from newspapers and financial market data in a daily measure of economic activity for Switzerland.

In what follows, we describe the data and methodology. Then, we provide an analysis of the in- and out-of-sample performance. The last section concludes.

Data

We use publicly available bond yields underlying the SIX Swiss Bond Indices® (SIX, 2020a). These data are available on a daily basis and with a delay of one day. Because many bond yields start only around 2007, we extend the series with a close match of government and corporate bond yields from the Swiss National Bank (see

[2] We plan to continuously extend the indicator. We therefore welcome suggestions for improvements and extensions.

Table A.2 and Figure A.2 in the Online Appendix).[3] Then, we compute various spreads that should be correlated with economic activity: a government bond term spread (8Y - 2Y), the interest rate differential vis-à-vis the euro area (1Y), and risk premia of short- and long-term corporate debt. Besides interest rate spreads for Switzerland, we compute risk premia of foreign companies that issue debt in Swiss franc for short- and long-term debt. We also include term spreads for the U.S. and for the euro area. For the latter, we use short-term interest rates in euro (European Central Bank, 2020) and long-term yields of German government debt (Deutsche Bundesbank, 2020). In addition, we include two implied volatility measures of the Swiss and U.S. stock market. Swiss data stem from SIX (2020b) and are published with a delay of one day. U.S. data stem from the Chicago Board Options Exchange (2020).

These financial market data should be related to the Swiss business cycle. Stuart (2020) shows that the term spread exhibits a lead on the Swiss business cycle.[4] Kaufmann (2020) argues that a narrowing of the interest rate differential appreciates the Swiss franc and thereby dampens economic activity. Risk premia are correlated with the default risk of companies, which should increase during economic crises. Finally, recent research documents an increase in uncertainty during economic downturns (Baker et al., 2016; Scotti, 2016). There are various ways to measure uncertainty (see e.g. Dibiasi and Iselin, 2016). Because we aim to exploit quickly and freely available financial market data, we prefer a measure of stock market volatility.

We complement the financial market data with sentiment indicators based on Swiss newspapers. We extract headlines and lead texts from the online archives of *Tages-Anzeiger*, the *Neue Zürcher Zeitung* and the *Finanz und Wirtschaft*.[5] We focus on the headline and lead text as these are publicly available and often contain the key messages of the articles. To reduce the number of potentially relevant articles, and to decompose the sentiment indicator into a domestic and foreign part, we only use articles satisfying specific search queries (See Table A.3 in the Online Appendix for a detailed description).

To calculate a news sentiment we use the lexical methodology (see, e.g., Ardia et al., 2019; Shapiro et al., 2017; Thorsrud, 2020). First, we filter out irrelevant information.[6] Second, we identify positive and negative words using the lexicon developed by Remus et al. (2010). Finally, we calculate for each article n and each day t a sentiment score:

$$S_{t,n} = \frac{\#P_{t,n} - \#N_{t,n}}{\#T_{t,n}} ,$$

[3] Data from the Swiss National Bank are published with a longer delay. Therefore, these bond yields cannot be used to track the economy on a daily basis.
[4] We therefore move forward all term spreads by half a year.
[5] During the first month of daily updates we noticed that the *Tages-Anzeiger* updates its archive with a relevant delay or not at all. For this in the revised version of the indicator we additionally include articles from the *Tages-Anzeiger* website.
[6] We remove HyperText Markup Language (HTML) tags, punctuation, numbers, and so-called stop words (e.g. the German words *der*, *wie*, *ob*). The stop words are provided by Feinerer and Hornik (2019). Also, we transform all letters to lowercase.

where $\#P_{t,n}, \#N_{t,n}, \#T_{t,n}$ represent, for each article and each time period, the number of positive, negative, and total words, respectively. Finally, we compute a simple average over all articles to obtain daily indicators for articles about the domestic and foreign economy.

News sentiment indicators receive more and more attention for forecasting economic activity. Buckman et al. (2020) show that during the Covid–19 pandemic, news sentiment indicators provide reliable and early information on the economy, even compared to quickly available survey data. Moreover, Ardia et al. (2019) show that news sentiment helps forecast U.S. industrial production growth.

Methodology

The financial market data and news indicators are quite volatile, but also they are correlated with each other. To parsimoniously summarize the information content of the data, and remove idiosyncratic noise, we estimate a factor model in static form:[7]

$$X = F\Lambda + e$$

The model comprises N variables and T daily observations. Therefore, the data matrix X is $(T \times N)$, the common factors F are $(T \times r)$, the factor loadings Λ are $(r \times N)$, and the unexplained error term e is $(T \times N)$. The advantage of a factor model is that we can parsimoniously summarize the information content in the large data matrix X with a relatively small number of common factors r. Assuming that the idiosyncratic components are only weakly serially and cross-sectionally correlated, we can estimate the factors and loadings by principal components (Bai and Ng, 2013; Stock and Watson, 2002).[8] Our main indicator is the first principal component of the static factor model. We normalize the indicator that it increases during crises.[9]

Because this factor has no clear economic interpretation, we decompose it into a contribution from domestic and foreign fluctuations. Suppose that there are only two factors driving the variables. One factor captures foreign fluctuations. The other factor captures domestic fluctuations. We allow for spillovers from abroad to the domestic economy, but not vice versa. Under these assumptions, the factor model reads:

---

[7] The news indicators are much more volatile than the financial market data (see Figure A.1 in the Online Appendix). We therefore compute a one-sided two-day moving average before including them in the factor model.

[8] To account for missing values, we compute the indicator only if at least five underlying data series are observed. Moreover, we remove all weekends. Then, we interpolate few additional missing values using an EM-algorithm (Stock and Watson, 2002), after normalizing the data to have zero mean and unit variance. For interpolation, we choose a relatively large number of factors for interpolating the data ($r = 4$). Finally, we estimate the f–curve as the first principal component of the interpolated data set.

[9] An interesting extension would be to examine whether more than one factor comprises relevant information for Swiss economic activity. We leave this extension for future research.

$$\begin{bmatrix} X & X^* \end{bmatrix} = \begin{bmatrix} f & f^* \end{bmatrix} \begin{bmatrix} \lambda_{11} & 0 \\ \lambda_{21} & \lambda_{22} \end{bmatrix} + e$$

where X, X^* denote data matrices comprising domestic and foreign variables, respectively. In addition f, f^* represent the domestic and foreign factors and $\lambda_{11}, \lambda_{21}, \lambda_{22}$ are loading matrices.

To estimate this factor model, we can use an iterative procedure inspired by Boivin et al. (2009). First, we estimate the foreign factor only on foreign data. This imposes that foreign variables only load on the foreign factor. Second, we estimate the domestic factor on $\tilde{X}$, where

$$\tilde{X} = X - \lambda_{21} f^* \ ,$$

removes variation explained by the foreign factor. We can estimate λ_{21} for every indicator comprised in X in a regression on the domestic and foreign factor. Because this regression depends on the value of the domestic factor, we repeat this step 50 times (see Boivin et al., 2009; Kaufmann and Lein, 2013, for more details). Finally, we can estimate a decomposition by regressing the f–curve on the domestic and foreign factors. This procedure does not guarantee that the decomposition adds up exactly to the overall factor. However, the unexplained rest turns out to be relatively small. The decomposition involves additional estimation steps that may reduce the forecast accuracy; therefore, we only use this decomposition for the in-sample interpretation, but not for out-of-sample forecasting.

Analysis

The f–curve should primarily be used to quickly detect turning points of the business cycle. As such, it is correlated or leading many key macroeconomic variables (see Figure A.4 in the Online Appendix). In its current form, we have not optimized the indicator to track any particular measure of economic activity. We therefore first focus on the in-sample information content of the f–curve, highlighting that it is available earlier than most other leading indicators. For the sake of illustration, however, we additionally provide an evaluation of its pseudo out-of-sample performance for forecasting real GDP growth.

In-sample analysis

To compare the in-sample information content of the f–curve to other leading indicators we perform a cross-correlation test (see Neusser, 2016, Ch. 12.1).[10] Figure 2 shows a substantial correlation between the f–curve and many prominent leading indicators.[11] There is a coincident or leading relationship with the KOF Economic Barometer, SECO's Swiss Economic Confidence, the Organisation for Economic

[10]It is noteworthy that other indicators are estimated or smoothed such that they undergo substantial revisions over time; moreover, some of the indicators are published with significant delays (see Table A.1 in the Online Appendix); finally, some are based on lagged data (see, e.g., OECD, 2010).

[11]Figure A.3 in the Online Appendix provides plots of these indicators.

Co-operation and Development composite leading indicator (OECD CLI), and consumer confidence.[12] There is a coincident relationship with trendEcon's perceived economic situation. This daily indicator starts only in 2006, however. There is a significant lagging relationship with the SNB's Business Cycle Index. But this index is published with a relevant delay. Overall, these results suggest the f–curve provides sensible information comparable with other existing indicators. The key advantage of the f–curve is its prompt availability and that it is available on a longer time period.

Figure 2 Cross-correlation with other indicators. Cross correlation between the f–curve and other prominent leading and sentiment indicators. We aggregate all data either to quarterly frequency (consumer sentiment) or monthly frequency (remaining indicators). The dashed lines give 95% confidence intervals. A bar outside of the interval suggests a statistically significant correlation between the indicators at a lead/lag of s. Before computing the cross-correlation the series have been pre-whitened with an AR(p) model (see Neusser, 2016, Ch. 12.1). The lag order has been determined using the Bayesian Information Criterion. The only exception is the OECD CLI for which we used an AR(4) model.

Figure 3 Decomposition domestic and foreign variables. Decomposition of the f–curve into foreign factors, domestic factors, and an unexplained rest

(a) Decomposition into domestic and foreign factors

(b) Decomposition into domestic and foreign factors during the Covid–19 crisis

Another advantage is that we can decompose its fluctuations into domestic and foreign factors. Panel (a) of Figure 3 shows that the foreign contribution rises after the collapse of Lehman Brothers, but also, during the euro area debt crisis. By contrast, the domestic contribution rises after the removal of the minimum exchange rate in 2015, but also, during the Covid–19 crisis. Focusing on the Covid–19 crisis, panel (b) shows the indicator rose already in the last week of February, before the actual Covid–19 lockdown. It reaches a peak during the first week of the lockdown and gradually declines thereafter. About half of the increase in the indicator can be traced back to foreign developments. Although the domestic lockdown is important, the f–curve suggests the Swiss economy would have suffered even in the absence of these restrictions. During the last four weeks, the contribution from foreign variables declines. The domestic contribution, however, remains elevated. Therefore, while the negative foreign demand shock seems to become less important, the model suggests economic activity will remain subdued also due to domestic headwinds.

Pseudo out-of-sample evaluation

How reliable is the f–curve? To answer this question we perform a pseudo real-time forecast evaluation. Therefore, we use the real-time data set for quarterly GDP

[12] All data sources are given in the Online Appendix.

vintages by Indergand and Leist (2014).[13] In the evaluation, we use the following direct forecasting model:

$$y_{\tau+h} = \alpha_h + \beta_{h,1} f_{\tau|t} + \beta_{h,2} f_{\tau-1} + \nu_{\tau+h}$$

where y_τ denotes quarterly GDP growth, h is the forecast horizon, τ gives time in quarterly frequency, and t denotes time in daily frequency. $f_{\tau|t}$ is our best guess of the f–curve for the entire quarter based on daily information at time t. We compute $f_{\tau|t}$ and f_τ as the simple average of available daily observations for a given quarter. Finally, $\nu_{\tau+h}$ is an error term. At the time of our last update, τ = 2020 Q2 and t = 4 June 2020.

We then conduct a forecast based on the state of information when a new quarterly GDP vintage is published by SECO.[14] This yields 70 nowcasts (69 one-quarter-ahead forecasts). These forecasts are compared to three benchmarks. First, we compare the forecasts to the first quarterly release of GDP growth for the corresponding quarter. Because quarterly GDP is substantially revised ex-post, we treat the initial quarterly GDP release as a forecast of the true GDP figure. Second, we use an autoregressive model of order 1, AR(1), estimated on the corresponding real-time vintage for GDP growth. Third, using the same forecasting equation as for the f–curve, we forecast GDP growth using the KOF Economic Barometer, a prominent monthly composite leading indicator (Abberger et al., 2014). To compute the forecast errors, we use the last available release of quarterly GDP from 3 June 2020.

Table 1 panel (a) shows the root-mean-squared error (RMSE) of the f–curve is higher than the one of the first official GDP release. However, the difference is not statistically significant. The advantage of the f–curve is, of course, that its value for the entire quarter is available about 2 months earlier than the first GDP release. In addition, we compare the f–curve to an AR(1) model. Panel (b) shows we outperform the AR(1) benchmark. The RMSE is 18% lower for the current quarter. Moreover, the difference in forecast accuracy is statistically significant. For the next quarter, however, the f–curve does not provide a more accurate forecast than the AR(1) model. Panel (c) shows that the f–curve yields similar results as the KOF Economic Barometer. The difference in the RMSE is never statistically significant. This suggest the advantage of our indicator primarily lies in its prompt availability.

We perform a subsample analysis in Table 2. The current vintage of GDP, which we use to compute the forecast errors, will likely be revised in the future. One of

[13]The evaluation is not strictly a real-time forecast evaluation because we use three types of in-sample information. First, the f–curve is constructed based on knowledge of the business cycle in the past, in particular, the Global Financial Crisis. Second, the link of the underlying indicators with new data is based on inspecting whether different data sources are highly correlated. Third, the normalization of the indicators in the factor model may introduce revisions that we do not account for in the forecast evaluation. Arguably, using this in-sample information in the evaluation makes sense if the goal of the evaluation is to show whether the indicator is useful going forward, rather than whether the indicator would have been useful in the past.

[14]These dates stem from Indergand and Leist (2014).

Table 1 Pseudo real-time evaluation. Root-mean-squared errors (RMSE) for forecasts on days with a new quarterly GDP release. A lower RMSE implies higher predictive accuracy. $h = 0$ ($h = 1$) denotes the forecast for the current (next) quarter. We use three benchmarks. First, we use the first quarterly release of the corresponding quarter (panel a). Second, we use an AR(1) model (panel b). Third, we use the KOF Economic Barometer (panel c). The Diebold-Mariano-West (DMW) test provides a p-value for the null hypothesis of equal predictive accuracy against the alternative written in the column header (Diebold and Mariano, 2002; West, 1996). We assume a quadratic loss function.

(a) Real GDP growth: First release vs. f–curve

	RMSE First release	RMSE f–curve	Relative RMSE first release/f–curve	DMW test (p-value) First release $<$ f–curve
$h = 0$	0.45	0.57	0.79	0.177
$h = 1$	0.45	0.7	0.64	0.042

(b) Real GDP growth: f–curve vs. AR(1)

	RMSE f–curve	RMSE AR(1)	Relative RMSE f–curve/AR(1)	DMW test (p-value) f–curve$<$ AR(1)
$h = 0$	0.57	0.7	0.82	0.039
$h = 1$	0.7	0.7	0.99	0.458

(c) Real GDP growth: f–curve vs. KOF Economic Barometer

	RMSE f–curve	RMSE Barometer	Relative RMSE f–curve/Barometer	DMW test (p-value) f–curve$<$ Barometer
$h = 0$	0.57	0.6	0.95	0.162
$h = 1$	0.7	0.65	1.07	0.832

Table 2 Subsample evaluation for real GDP growth: First release vs. f–curve. Root-mean-squared errors (RMSE) for forecasts on days with a new quarterly GDP release. A lower RMSE implies higher predictive accuracy. $h = 0$ ($h = 1$) denotes the forecast for the current (next) quarter. Panel (a) shows the evaluation for GDP figures that include the annual SFSO estimates (until 2018). Panel (b) excludes economic crises. As benchmark we use the first quarterly release of the corresponding quarter. The Diebold-Mariano-West (DMW) test provides a p-value for the null hypothesis of equal predictive accuracy against the alternative written in the column header (Diebold and Mariano, 2002; West, 1996). We assume a quadratic loss function.

(a) Only when annual GDP estimates available (2000-2018)

	RMSE First release	RMSE f–curve	Relative RMSE first release/f–curve	DMW test (p-value) First release $<$ f–curve
$h = 0$	0.47	0.46	1.03	0.605
$h = 1$	0.46	0.6	0.77	0.061

(b) Without crises (excluding 2008, 2009, 2020)

	RMSE First release	RMSE f–curve	Relative RMSE first release/f–curve	DMW test (p-value) First release $<$ f–curve
$h = 0$	0.4	0.4	1	0.504
$h = 1$	0.4	0.43	0.91	0.166

the reasons is that future vintages will include annual GDP estimates by the SFSO, which are based on comprehensive firm surveys. Therefore, we restrict the sample to years where the GDP figures already include these annual figures (panel a). The f–curve performs better on this sample. In fact, the RMSE is almost identical to the RMSE of the first GDP release for the current quarter. A similar picture emerges when excluding economic crises (panel b). This implies that the f–curve does not only signal deep economic crises, but tracks the economy well also during normal times.

Are the financial market or news data more important for the forecasting performance of the f–curve? Figure 4 shows two indicators only calculated with financial market and news data, respectively. Although the indicators are positively correlated, there are two key differences. First, the financial market data respond more strongly during crises. Second, the news data are more volatile[15] This suggests the financial market data provide a more accurate signal of the business cycle than the news data. Table 3 confirms this view. The RMSE for an indicator based only on financial market variables amounts to 0.57, the same as for the overall f–curve. Meanwhile, the RMSE of a forecast based only on news data amounts to 0.64. The news data does not worsen the f–curve because the factor model including financial market data removes the idiosyncratic fluctuations; taken in isolation, however, the news indicator performs worse.

Figure 4 Comparison news and financial market data. Two indicators estimated only on financial market and news data, respectively.

Figure 5 Real-time results since initial version of the f–curve. Panel (a): average number of observations available for calculation the f–curve (left figure). The different shades of gray represent estimates over time from 11 May 2020 to 29 May 2020 (right figure). Panel (b): Estimates of the f–curve using the methodology in the Working Paper (v1.0) and the current version (v2.0).

(a) Real-time estimates during first month

(b) Working paper (v1.0) and published version (v2.0)

Although it is too early to judge the actual real-time performance of the indicator, Figure 5 provides some preliminary results on the stability of the f–curve over time. One reason why the indicator is revised is that not all data series are available in real-time (ragged edge problem). Panel (a) shows results over the first month we updated the indicator on a daily basis. On average, more than 8 out of 12 series

[15]This is also because we smooth the news indicator with a moving-average of only 2 days. Comparable studies smooth over a longer time period. For example, Thorsrud (2020) uses a moving average of 60 days. On the one hand, this reduces the volatility of the news sentiment. On the other hand, this obviously renders the indicator less useful for detecting rapid daily changes.

Table 3 Comparison news vs. financial data. Root-mean-squared errors (RMSE) for forecasts on days with a new quarterly GDP release. A lower RMSE implies higher predictive accuracy. $h = 0$ ($h = 1$) denotes the forecast for the current (next) quarter. Panel (a) shows the evaluation for an indicator based only on financial market data. Panel (b) shows the evaluation for an indicator based only on news data. As benchmark we use the first quarterly GDP release for the corresponding quarter. The Diebold-Mariano-West (DMW) test provides a p-value for the null hypothesis of equal predictive accuracy against the alternative written in the column header (Diebold and Mariano, 2002; West, 1996). We assume a quadratic loss function.

(a) Only financial market data

	RMSE<br>First release	RMSE<br>f–curve	Relative RMSE<br>first release/f–curve	DMW test (p-value)<br>First release < f–curve
$h = 0$	0.45	0.57	0.79	0.19
$h = 1$	0.45	0.71	0.63	0.042

(b) Only news data

	RMSE<br>First release	RMSE<br>f–curve	Relative RMSE<br>first release/f–curve	DMW test (p-value)<br>First release < f–curve
$h = 0$	0.45	0.64	0.71	0.047
$h = 1$	0.45	0.7	0.63	0.019

are available with a delay of one day. After three days, almost all indicators are available.

The main reason why the average lies below 12 is that the archive of *Tages-Anzeiger* has not been updated since 12 May 2020.[16] Therefore, we augmented the indicator with information from this newspapers' online edition. Adding this source resulted in a slightly larger revision of the indicator compared to the Working Paper version (see Panel b). However, the correlation between the old and new version is 0.99 and the broad picture during the Covid–19 crisis is identical.

Concluding remarks

We develop a daily indicator of Swiss economic activity. A major strength of the Indicator is that it can be updated with a delay of only one day. An evaluation of the indicator shows that it is not only correlated with other business cycle indicators but also accurately tracks Swiss GDP growth. Therefore, the f–curve provides an accurate and flexible framework to track Swiss economic activity at high frequency.

Having said that, there is still room for improvement. We see six promising avenues for future research. First, the news sentiment indicators could exploit other publicly available news sources, in particular, newspapers from the French- and Italian-speaking parts of Switzerland. Second, we could use a topic modeling algorithm, instead of our own search queries, to classify news according to countries, sectors, and economic concepts (see e.g. Thorsrud, 2020). Third, the lexicon could be tailored specifically to economic news (see e.g. Shapiro et al., 2017). Fourth, we could examine the predictive ability of multiple factors and for other macroeconomic data. Fifth, the information could be used to disaggregate quarterly GDP and industrial production into monthly or even weekly series. Finally, it would be desirable to collect and exploit the information from many different daily indicators that are currently developed into one single composite indicator or indicator data

[16] On rare occasions, the websites of other sources were not available.

set. Exploiting all this new information will likely further improve our understanding of health of the Swiss economy at high frequency.

List of abbreviations

AR(p) Autoregressive model of order p
CH Switzerland
CLI Composite leading indicator
Covid−19 Coronavirus disease of 2019
EUR Europe / Euro
***f*−curve** Fever curve
FuW *Finanz und Wirtschaft*
GDP Gross domestic product
HTML HyperText Markup Language
ILO International Labor Organization
KOF *Konjunkturforschungsstelle*
NZZ *Neue Zürcher Zeitung*
OECD Organisation for Economic Co-operation and Development
Q Quarter
RMSE Root-mean-squared error
SECO State Secretariat for Economic Affairs
SFSO Swiss Federal Statistical Office
SNB Swiss National Bank
TA *Tages-Anzeiger*
Y Year

Data availability
Data are available on github.com/dankaufmann/f-curve/.

Competing interests
The authors declare that they have no competing interests.

Funding
Not applicable.

Authors' contributions
Not applicable.

Acknowledgments
We thank an anonymous referee, Ronald Indergand, Alexander Rathke, and Jan-Egbert Sturm for helpful discussions.

Author details
[1]Institute of Economic Research, University of Neuchâtel, Rue A.-L. Breguet 2, 2000 Neuchâtel, Switzerland. [2]KOF Swiss Economic Institute, ETH Zurich, Zurich, Switzerland.

References

Abberger, K., Graff, M., Siliverstovs, B., Sturm, J.-E.: The KOF Economic Barometer, Version 2014. A composite leading indicator for the Swiss business cycle. KOF Working Papers 353, KOF Swiss Economic Institute, ETH Zurich (2014). doi:10.3929/ethz-a-010102658

Ardia, D., Bluteau, K., Boudt, K.: Questioning the news about economic growth: Sparse forecasting using thousands of news-based sentiment values. International Journal of Forecasting **35**(4), 1370–1386 (2019). doi:10.1016/j.ijforecast.2018.10.010

Bai, J., Ng, S.: Principal components estimation and identification of static factors. Journal of Econometrics **176**(1), 18–29 (2013). doi:10.1016/j.jeconom.2013.03.007

Baker, S.R., Bloom, N., Davis, S.J.: Measuring economic policy uncertainty. The Quarterly Journal of Economics **131**(4), 1593–1636 (2016). doi:10.1093/qje/qjw024

Becerra, A., Eichenauer, V.Z., Indergand, R., Legge, S., Martinez, I., Mühlebach, N., Oguz, F., Sax, C., Schuepbach, K., Thöni, S.: trendEcon. Accessed: 30/04/2020 (2020). https://www.trendecon.org

Boivin, J., Giannoni, M.P., Mihov, I.: Sticky prices and monetary policy: Evidence from disaggregated us data. American Economic Review **99**(1), 350–84 (2009). doi:10.1257/aer.99.1.350

Brown, M., Fengler, M.: MonitoringConsumptionSwitzerland. Accessed: 03/05/2020 (2020). https://public.tableau.com/profile/monitoringconsumptionswitzerland

Buckman, S.R., Shapiro, A.H., Sudhof, M., Wilson, D.J.: News sentiment in the time of COVID-19. FRBSF Economic Letter **2020**(08), 1–05 (2020). Accessed: 13/05/2020

Chicago Board Options Exchange: CBOE Volatility Index: VIX [VIXCLS]. Accessed: 30/04/2020 (2020). https://fred.stlouisfed.org/series/VIXCLS

Deutsche Bundesbank: Zeitreihe BBK01.WT1010: Rendite der jeweils jüngsten Bundesanleihe mit einer vereinbarten Laufzeit von 10 Jahren. Accessed: 30/04/2020 (2020). https://www.bundesbank.de/dynamic/action/de/statistiken/zeitreihen-datenbanken/zeitreihen-datenbank/723452/723452?tsId=BBK01.WT1010

Dibiasi, A., Iselin, D.: Measuring uncertainty. KOF Bulletin 101, KOF Swiss Economic Institute, ETH Zurich (2016). Accessed: 30/04/2020. https://ethz.ch/content/dam/ethz/special-interest/dual/kof-dam/documents/KOF_Bulletin/kof_bulletin_2016_11_en.pdf

Diebold, F.X., Mariano, R.S.: Comparing predictive accuracy. Journal of Business & economic statistics **20**(1), 134–144 (2002). doi:10.1198/073500102753410444

Eckert, F.: A mobility indicator for Switzerland. Accessed: 14/05/2020 (2020). https://kof.ethz.ch/en/news-and-events/news/kof-bulletin/kof-bulletin/2020/05/ein-mobilitaetsindikator-fuer-die-schweiz.html

European Central Bank: Yield curve spot rate, 1-year maturity - Government bond, nominal, all issuers whose rating is triple . Accessed: 30/04/2020 (2020). https://sdw.ecb.europa.eu/browseExplanation.do?node=qview&SERIES_KEY=165.YC.B.U2.EUR.4F.G_N_A.SV_C_YM.SR_1Y

Feinerer, I., Hornik, K.: Tm: Text Mining Package. (2019). R package version 0.7-7. https://CRAN.R-project.org/package=tm

Galli, A.: Which indicators matter? Analyzing the Swiss business cycle using a large-scale mixed-frequency dynamic factor model. Journal of Business Cycle Research **14**(2), 179–218 (2018). doi:10.1007/s41549-018-0030-4

Indergand, R., Leist, S.: A real-time data set for Switzerland. Swiss Journal of Economics and Statistics **150**(IV), 331–352 (2014). doi:10.1007/BF03399410

Kaufmann, D.: Wie weiter mit der Tiefzinspolitik? Szenarien und Alternativen. IRENE Policy Reports 20-01, IRENE Institute of Economic Research (April 2020). https://ideas.repec.org/p/irn/polrep/20-01.html

Kaufmann, D., Lein, S.M.: Sticky prices or rational inattention – what can we learn from sectoral price data? European Economic Review **64**, 384–394 (2013). doi:10.1016/j.euroecorev.2013.10.001

Kaufmann, D., Scheufele, R.: Business tendency surveys and macroeconomic fluctuations. International Journal of Forecasting **33**(4), 878–893 (2017). doi:10.1016/j.ijforecast.2017

Lewis, D.J., Mertens, K., Stock, J.H.: Monitoring real activity in real time: The weekly economic index. Liberty Street Economics 30/03/2020, Federal Reserve Bank of New York (March 2020). Accessed: 13/05/2020. https://libertystreeteconomics.newyorkfed.org/2020/03/monitoring-real-activity-in-real-time-the-weekly-economic-index.html

Neusser, K.: Time Series Econometrics, pp. 207–214. Springer, Cham (2016). doi:10.1007/978-3-319-32862-1_11

OECD: Review of the CLI for 8 countries. OECD Composite Indicators (2010). Accessed: 13/05/2020

Remus, R., Quasthoff, U., Heyer, G.: SentiWS - a publicly available German-language resource for sentiment analysis. In: Proceedings of the Seventh International Conference on Language Resources and Evaluation (LREC'10). European Language Resources Association (ELRA), Valletta, Malta (2010)

Scotti, C.: Surprise and uncertainty indexes: Real-time aggregation of real-activity macro-surprises. Journal of Monetary Economics **82**(C), 1–19 (2016). doi:10.1016/j.jmoneco.2016.06.002

Shapiro, A.H., Sudhof, M., Wilson, D.J.: Measuring news sentiment. Working Paper Series 2017-1, Federal Reserve Bank of San Francisco (January 2017). doi:10.24148/wp2017-01

SIX: SBI®–Swiss Bond Indices. Accessed: 30/04/2020 (2020a). https://www.six-group.com/exchanges/indices/data_centre/bonds/sbi_en.html

SIX: VSMI®–Volatility Index on the SMI®. Accessed: 30/04/2020 (2020b). https://www.six-group.com/exchanges/indices/data_centre/strategy_indices/vsmi_en.html

Stock, J.H., Watson, M.W.: Macroeconomic forecasting using diffusion indexes. Journal of Business & Economic Statistics **20**(2), 147–162 (2002). doi:10.1198/073500102317351921

Stuart, R.: The term structure, leading indicators, and recessions: evidence from Switzerland, 1974–2017. Swiss Journal of Economics and Statistics **156**(1), 1–17 (2020). doi:10.1186/s41937-019-0044-4

Thorsrud, L.A.: Words are the new numbers: A newsy coincident index of the business cycle. Journal of Business & Economic Statistics **38**(2), 393–409 (2020). doi:10.1080/07350015.2018.1506344

Wegmüller, P., Glocker, C.: 30 Indikatoren auf einen Schlag. Die Volkswirtschaft, 19–22 (2019)

West, K.: Asymptotic inference about predictive ability. Econometrica **64**(5), 1067–84 (1996). doi:10.2307/2171956

Additional Files

Online Appendix

The Online Appendix to this paper is available on www.dankaufmann.com/publications/

Replication files

Codes for replication of the main indicator are available on github.com/dankaufmann/f-curve/
